# Shifting patterns of genomic variation in the somatic evolution of papillary thyroid carcinoma

**DOI:** 10.1186/s12885-016-2665-7

**Published:** 2016-08-18

**Authors:** Jill C. Rubinstein, Taylor C. Brown, Emily R. Christison-Lagay, Yawei Zhang, John W. Kunstman, C. Christofer Juhlin, Carol Nelson-Williams, Gerald Goh, Courtney E. Quinn, Glenda G. Callender, Robert Udelsman, Richard P. Lifton, Reju Korah, Tobias Carling

**Affiliations:** 1 Yale Endocrine Neoplasia Laboratory, Yale University School of Medicine, New Haven, 06520 Connecticut USA; 2 Department of Surgery, Yale University School of Medicine, New Haven, 06520 Connecticut USA; 3 Department of Environmental Health Sciences, School of Public Health, Yale University School of Medicine, New Haven, 06520 Connecticut USA; 4 Department of Genetics, Yale University School of Medicine, New Haven, 06520 Connecticut USA; 5 Howard Hugh Medical Institute, Chevy Chase, Maryland USA; 6 Yale School of Medicine, 333 Cedar Street, FMB130A, P.O. Box 208062, New Haven, CT 06520 USA; 7 Present Address: Department of Oncology-Pathology (OnkPat), Karolinska Universitetssjukhuset, Solna, 171 76 Stockholm Sweden; 8 Present Address: UCL Cancer Institute, University College London, London, UK

**Keywords:** Somatic evolution, Papillary thyroid cancer, Exome sequencing

## Abstract

**Background:**

Cancer is increasingly understood to arise in the context of dynamically evolving genomes with continuously generated variants subject to selective pressures. Diverse mutations have been identified in papillary thyroid carcinoma (PTC), but unifying theories underlying genomic change are lacking. Applying a framework of somatic evolution, we sought to broaden understanding of the PTC genome through identification of global trends that help explain risk of tumorigenesis.

**Methods:**

Exome sequencing was performed on 53 PTC and matched adjacent non-tumor thyroid tissues (ANT). Single nucleotide substitution (SNS) signatures from each sample pair were divided into three subsets based on their presence in tumor, non-tumor thyroid, or both. Nine matched blood samples were sequenced and SNS signatures intersected with these three subsets. The intersected genomic signatures were used to define branch-points in the evolution of the tumor genome, distinguishing variants present in the tissues’ common ancestor cells from those unique to each tissue type and therefore acquired after genomic divergence of the tumor, non-tumor, and blood samples.

**Results:**

Single nucleotide substitutions shared by the tumor and the non-tumor thyroid were dominated by C-to-T transitions, whereas those unique to either tissue type were enriched for C-to-A transversions encoding non-synonymous, predicted-deleterious variants. On average, SNSs of matched blood samples were 81 % identical to those shared by tumor and non-tumor thyroid, but only 12.5 % identical to those unique to either tissue. Older age and BRAF mutation were associated with increased SNS burden.

**Conclusions:**

The current study demonstrates novel patterns of genomic change in PTC, supporting a theory of somatic evolution in which the zygote’s germline genome undergoes continuous remodeling to produce progressively differentiated, tissue-specific signatures. Late somatic events in thyroid tissue demonstrate shifted mutational spectra compared to earlier polymorphisms. These late events are enriched for predicted-deleterious variants, suggesting a mechanism of genomic instability in PTC tumorigenesis.

**Electronic supplementary material:**

The online version of this article (doi:10.1186/s12885-016-2665-7) contains supplementary material, which is available to authorized users.

## Background

Papillary Thyroid Carcinoma (PTC) is the most common type of thyroid cancer, accounting for more than 75 % of all thyroid malignancies. The incidence of PTC is increasing more rapidly than any other cancer [1]. Previous studies have noted that PTC carries a low overall burden of genomic insult relative to anaplastic thyroid cancer and other malignancies [2, 3]. Several mutually exclusive, recurrent genomic events have been identified, including BRAF and RAS mutations and RET- and NTRK1- gene fusions which are found in up to 70 % of tumors [4, 5]. Mutations in the phosphoinositide 3-kinase (PI3K) pathway genes PTEN, PIK3CA, and AKT1 have been reported at lower frequencies as well as alterations in the EIF1AX, PPM1D, and CHEK2 genes [2, 6, 7]. This increasingly comprehensive catalog of recurrent genomic events allows for more specific tumor sub-classification and may provide clues to molecular mechanisms driving tumorigenesis. Despite such a diversity of genomic alterations, PTC does not demonstrate great clinical heterogeneity, suggesting that a unifying concept of the genomic contribution to malignant transformation continues to be elusive. In an evolutionary view of cancer, tumorigenesis can be seen in terms of genomic instability causing stochastic accumulation of variants. Those acquired variants providing a selective advantage at the cellular level will be propagated, resulting in stepwise accumulation of diverse genomic alterations, and ultimately tumors with highly individualized genotypes [8].

The availability of genome-wide data at single-nucleotide resolution has spurred interest in the somatic evolution model, with clonal expansions of cells arising in the context of continuous variant generation and natural selection [9–12]. A recent study correlating tissue-specific cancer risk with the number of stem cell divisions highlighted the importance of the accumulation of stochastic change introduced during successive cycles of DNA replication [13]. Takeda et al. used a mouse model to investigate heterogeneity resulting from an evolving genome, searching for drivers appearing early in tumors’ phylogenetic trees, thereby making them more widely distributed within the tumors [14]. Sequencing of distinct regions within individual lung tumors demonstrated spatial separation of drivers, showing evolutionary divergence of clones with concomitant shifts in mutational spectra [15]. An accompanying study found that primary lung tumors with larger fractions of sub-clonal mutations have increased likelihood of relapse [16]. Finally, studying myeloproliferative neoplasms, Ortmann et al. demonstrated that the order in which mutations occur during clonal evolution plays a critical role in tumor behavior [17].

The growing number of studies exploring an evolutionary model of cancer provides a framework to explain the sequential acquisition of tumor behaviors (eg. evasion of immune surveillance, metastatic ability, resistance to therapy) and highlights some of the challenges faced in attempting to design tailored therapies [11, 12, 14]. Unlike highly aggressive tumors which demonstrate rapid growth and require multiple chemotherapeutic (and frequently surgical) interventions, PTC’s indolent nature and relatively low mutational burden makes it an ideal candidate for the study of the sequential acquisitions of genomic variations.

This study explores the hypothesis that a rudimentary phylogenetic tree can be constructed for each tumor by exploring three subsets of single nucleotide substitutions (SNS): those present in tumor alone, those present in adjacent non-tumor tissue alone, or those present in both and therefore presumed to reflect the germline or variants accrued early in embryogenesis. Paired blood samples from a subset of patients were also examined to strengthen the hypothesis that SNS signatures of multiple tissues from a single patient, while sharing a great deal of homology derived from their common germline, also demonstrate tissue specific patterns of genomic variation. A more critical examination of the SNSs in each group yields insight into the topography of the PTC mutational signature.

## Methods

### Patient information and tissue samples

DNA from 53 fresh-frozen papillary thyroid tumors and paired non-tumor thyroid tissues resected between 2000 and 2011, for which sufficient quantities of tissue were available, were identified in the local biobank. Diagnosis was confirmed by a pathologist and TNM stages were determined using World Health Organization criteria. DNA was extracted using AllPrep DNA Mini Kit and following the protocol provided by the manufacturer (Qiagen, MD, USA). DNA from nine available matched blood samples was extracted using QIAamp DNA Mini Kit per manufacturer protocol (Qiagen, MD, USA). Patient demographic information is listed in Additional file 1: Table S1.

### Exome sequencing

Genomic DNA from fresh-frozen matched pairs of tumor, corresponding adjacent non-tumor thyroid tissue, and blood samples was sequenced at the Yale Center for Genome Analysis following an internal protocol using NimbleGen v2.0 exome capture reagent (Roche) and sequenced through Illumina HiSeq 2000, 75 base-paired end reads. Reads were subsequently mapped to reference genome hg19 using ELANDv2, single nucleotide variants assigned a quality score (QS) using SAMtools, filtered with bcfTools varFilter using default parameters. Sequencing yielded and average of 95 million and 198 million reads per lane, for non-tumor and tumor tissue, respectively. 20x coverage was achieved for 92 % of non-tumor bases and 95 % of tumor sample bases. A minimum of 8x coverage was required for inclusion and standard quality score cut-offs werre employed. Samples were then annotated using ANNOVAR (www.openbioinformatics.org/annovar) and filtered to remove variants present in dbSNP, build 129 [18]. Additional filters were included for alternate analyses, including dbSNP build 135, and the 1000 Genomes SNP filter. Additionally, 53 race-matched samples that had undergone exome sequencing on the Illumina platform were chosen at random from the 1000 Genomes project and processed identically [19]. Computational prediction of the likelihood that each SNS would result in a deleterious change to the protein product was performed using 5 independent algorithms (PolyPhen-2, MutationTaster, SIFT, radialSvm, lr) as implemented in the ANNOVAR package.

SNS signatures from each PTC were intersected with those from their matched ANT to define subsets of SNSs shared between the tissues (Common subset), and those present in one tissue-type but not the other (Unique-to-PTC, Unique-to-ANT). The nine matched blood samples were intersected with each corresponding PTC/ANT subset individually. ConsensusPathDB [20, 21] pathway enrichment analysis was performed for each subset using all genes containing at least one non-synonymous, stopgain, or stoploss variant. MAPK pathway genes were downloaded from the KEGG database (www.genome.jp/kegg/kegg1.html). RET-PTC fusions were detected with RT-PCR. RNA was isolated using AllPrep DNA/RNA/Protein Mini Kit, cDNA created with iScript cDNA Synthesis Kit, and PCR amplification performed (primers: RET/PTC1 forward primer 5-ATT GTC ATC TCG CCG TTC-3 (H4 domain), RET/PTC1 reverse primer 5-TGC TTC AGG ACG TTG AAC-3, RET/PTC3 forward primer 5-TGGAGA AGA GAG GCT GTA TC-3 (RFG domain), RET/PTC3 reverse primer 5- CGT TGC CTT GAC TTT TC-3). RET/PTC translocations were identified as 306 and 268 base pair amplicons for RET/PTC1 and RET/PTC3, respectively. TERT promoters underwent Sanger sequencing at the Keck DNA Sequencing Facility. All in house computational analyses utilized the R Project for Statistical Computing with statistical testing via Welch two sample *t*-test and ANOVA.

## Results and discussion

### Study cohort and sequencing summary

The study cohort included 53 PTC patients with a mean age at the time of surgery of 48.9 years (range 16-97) and a female to male ratio of 2.5 (see Additional file 1: Table S1 for demographic and clinical characteristics). Exome sequencing of genomic DNA from 53 matched PTCs and matched adjacent non-tumor thyroid samples (ANTs) yielded a mean 198- and 95-fold coverage, respectively, with greater than 8x coverage in over 96 % of bases. Tumor purity ranged from 41.1 to 69.5 %.

Analysis of the 53 PTC specimens confirmed the presence of previously identified recurrent alterations, including BRAF (41.5 %), TERT (5.7 %), and RET-PTC fusions (3.8 %). PTCs with BRAF mutations had greater SNS counts than those without (mean 6996 vs. 5976, *p* < 0.001). Additionally, patients older than 45 years of age had more SNSs than those under forty-five (mean 6705 vs. 6014, *p* < 0.01). There was no significant difference in SNS count on the basis of gender, stage, or histological subtype (Additional file 1: Table S1).

### Intersected SNS signatures define late somatic changes

**Fig. 1:**
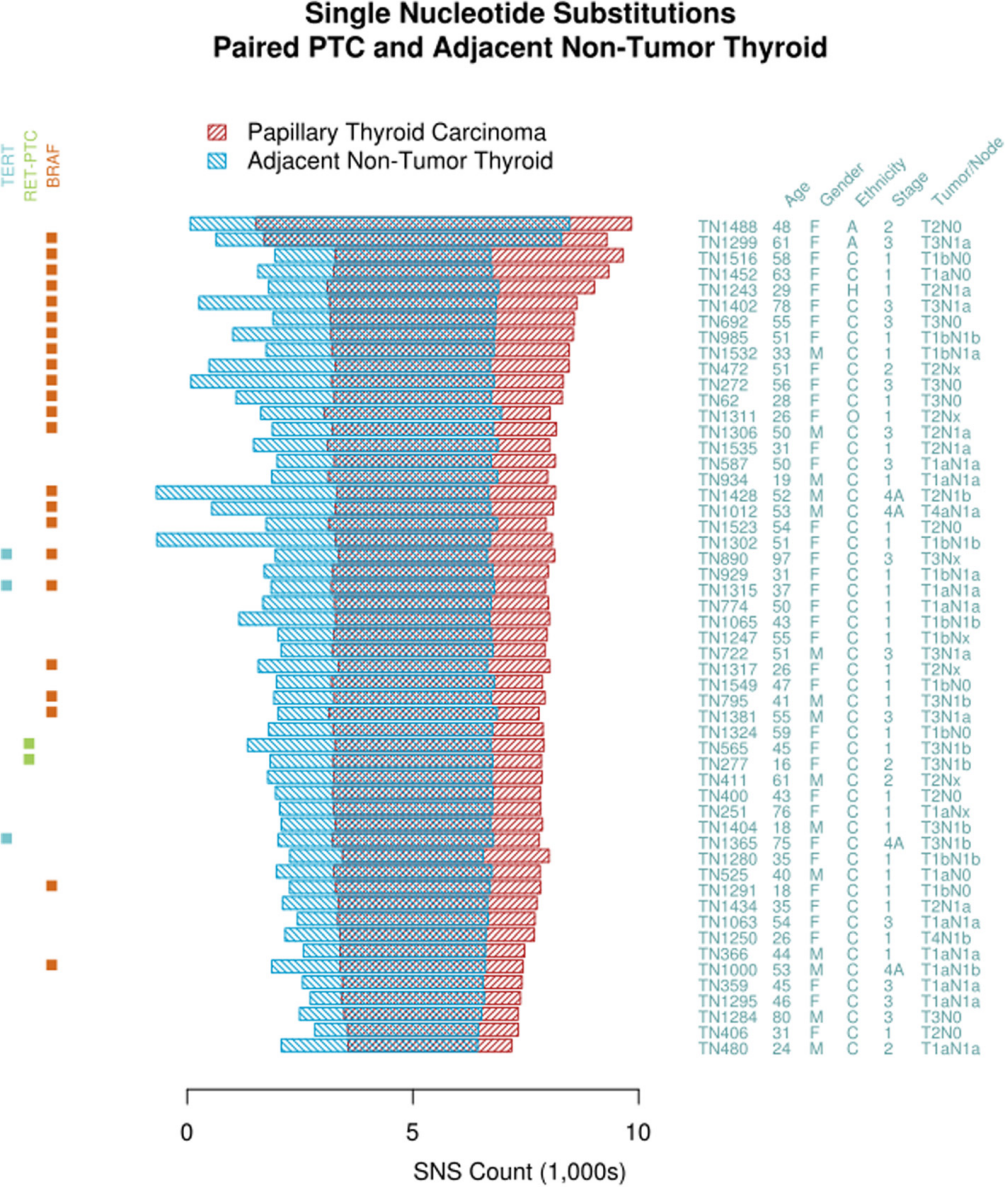
Single Nucleotide Substitution Signatures. Pairwise comparison of each PTC with its matched ANT defines three subsets of SNSs, those unique to the PTC (*right*), those unique to the ANT (*left*), and the Common subset (*middle*). PTCs share between 57 and 88 % (mean 76 %) of their SNSs with the background ANT in which they arise. BRAF-mutated PTCs and patients over 45 years of age have a greater number of SNSs. (M = Male, F = Female; A = African American, C = Caucasian, H = Hispanic, O = Other; TN refers to the AJCC TNM staging system)

Global SNS signatures for each paired PTC and ANT are shown in Fig. 1. Across the cohort, between 57 and 88 % of PTC SNSs also exist in the paired ANT. This shared portion (designated hereafter as the Common subset) represents SNSs present in the sample pair’s most recent common cellular ancestor, i.e. those present in the patient’s true germline (the zygote), together with those accrued during embryogenesis and thyroid differentiation, up to the point of divergence of the malignant from the non-malignant thyrocytes. The areas on either flank in the plot are SNSs accrued by the malignant and non-malignant sub-clones following divergence (designated hereafter as the Unique-to-PTC and Unique-to-ANT subsets).

**Fig. 2:**
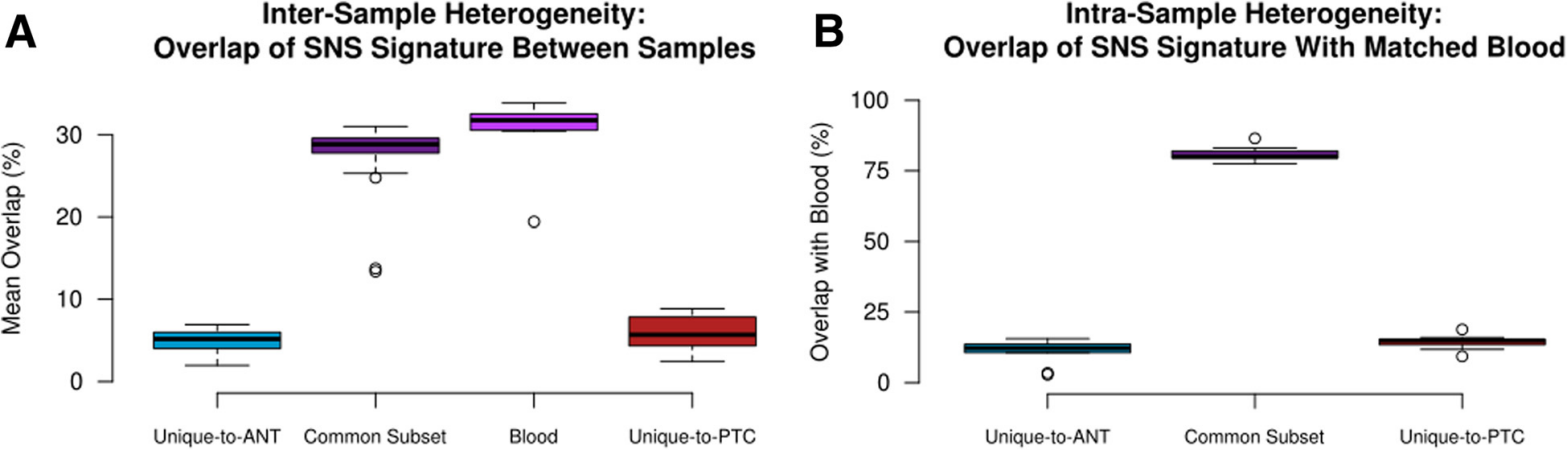
**a** Inter-sample heterogeneity. SNS signatures demonstrate a mean overlap of 28 % and 30.6 % between any two samples in the Common subset and blood samples. The low outliers in each of these groups represent samples from African-American patients. The Unique-to-ANT and Unique-to-PTC subsets have an even larger degree of diversity, sharing a mean of only 6.0 % and 4.9 % of their SNSs. **b** Comparison of each sample with its matched blood demonstrates that the majority of Common subset SNSs (mean 80.9 %) are also found in the blood samples. The Unique-to-ANT and Unique-to-PTC subsets share only 10.8 % and 14.3 % of SNSs with their matched blood

The accumulation of SNSs throughout the lifetime of the thyroid gland should be driven in large part by stochastic change introduced during sequential cycles of DNA replication and cell division within a greater framework mutational susceptibility [8]. Each thyroid gland (as well as every other tissue type) is therefore expected to follow a unique evolutionary pathway, with SNSs accrued at varying points in differentiation, resulting in large degrees of heterogeneity among tumors. Calculating the degree of SNS overlap of each PTC/ANT sample pair with every other sample confirms that on average only 28.0 % of each Common subset’s and 30.6 % of each blood sample’s SNSs are shared with another sample. Those SNSs Unique-to-PTC or Unique-to-ANT share only 6.0 % and 4.9 % of SNSs, respectively (Fig. 2a). For the nine patients with available blood samples, there was significantly greater overlap between the matched Common subset and blood SNSs than between the Unique-to-ANT or Unique-to-PTC subsets and the blood (mean 80.9 % vs. 10.8 % and 14.3 %, respectively; *p* < 0.0001), strengthening the argument that the Common subset SNSs consist mainly of germline variants plus those accrued earlier in embryogenesis, prior to divergence of hematopoietic from thyrocyte lineages (Fig. 2b).

### Shifting mutational spectra increase risk for deleterious change

**Fig. 3:**
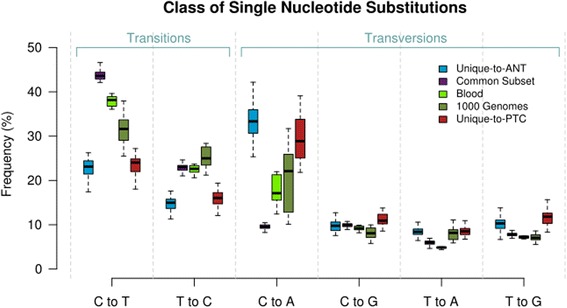
Mutational spectra. The SNS signatures of the Common subsets and the blood samples demonstrate a dominance of C-to-T transitions across the cohort. Those SNSs that are specific to the thyroid gland, the Unique-to-ANT and the Unique-to-PTC Subsets, demonstrate a significant shift toward C-to-A transversions

SNSs are divided into transitions (those exchanging a purine for purine or pyrimidine for pyrimidine) and transversions (those exchanging a purine for pyrimidine or pyrimidine for purine). Transitions are less likely than transversions to result in non-synonymous change in the final amino acid sequence and are more prevalent than transversions, partly due to frequent spontaneous deamination of 5-methylcytosine to thymine [22, 23]. Comparison of transition:transversion ratios (Tr:Tv) demonstrates that the Common subset consistently has the highest ratio, with a mean 3.4- and 3.2-fold increase over the Unique-to-ANT and Unique-to-PTC subsets, respectively (Additional file 2: Figure S1). Fig. 3 demonstrates the distribution of each transition and transversion for the Unique-to-ANT, Common, and Unique-to-PTC subsets as well as the matched blood samples and an independent cohort of blood samples from the 1000 Genomes Project. Those evolutionarily older SNSs (the Common subset) are dominated by C-to-T transitions, while those accrued after divergence of the thyroid-specific subsets (Unique-to-ANT and Unique-to-PTC) demonstrate a dramatic shift toward C-to-A transversions (*p* < 0.0001), suggesting that evolutionary forces at work on the genome of thyroid progenitor cells in the course of organogenesis differ from those seen later in the course of the gland’s development and during natural thyrocyte turnover. To ensure that the observed trend was not introduced through a filtering bias, the analysis was repeated with varying levels of SNS filtering. The shift in mutational spectra persisted regardless of the level of filtering (Additional file 3: Figure S2).

**Fig. 4:**
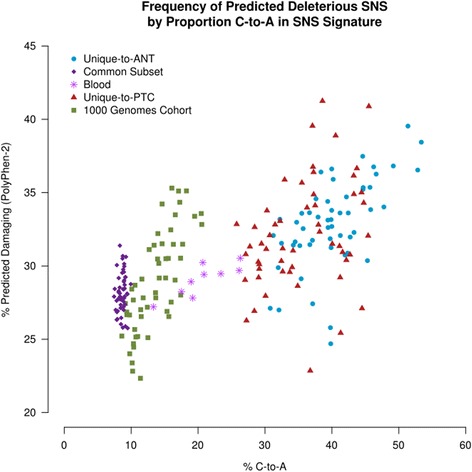
Predicted impact of SNS on protein function. Based upon computational prediction with PolyPhen-2, the observed shift toward increased numbers of C-to-A transversions is accompanied by an increased likelihood of accruing damaging variants in both the Unique-to-PTC and Unique-to-ANT Subsets (*p* < 0.0001)

There are 64 possible codons in the triplet human genetic code, but only 20 amino acids and 3 stop codons to be encoded. This redundancy is the mechanism through which some SNSs result in synonymous changes, whereas others lead to a change in the amino acid sequence. The frequency with which potential synonymous and non-synonymous changes occur differs among the different possible SNSs. For example, 34.4 % of all potential C-to-T SNSs in the genetic code result in synonymous changes, compared to only 19.8 % of all potential C-to-A substitutions (Additional file 4: Figure S3). As therefore expected, the observed shift in PTC mutational spectra at different points in the evolution of its genome is associated with increased non-synonymous change in both the Unique-to-ANT and Unique-to-PTC subsets (*p* < 0.0001) (Additional file 5: Figure S4). Computational assessment of the likelihood that these changes will impact protein function demonstrated increased numbers of predicted-deleterious variants in the Unique-to-ANT and Unique-to-PTC subsets relative to the Common subset, the blood samples, and the 1000 Genomes cohort (*p* < 0.0001) (Fig. 4, Additional file 6: Figure S5) [24]. Interestingly, both the malignant (Unique-to-PTC) and the non-malignant (Unique-to-ANT) subsets share this increased propensity to accumulate predicted-deleterious changes, suggesting that: 1) the global shift in mutational pattern is characteristic of the thyroid genome and does not reflect an evolutionary pressure introduced by the tumor; and 2) thyroid tissue appears more tolerant of mutation-prone SNSs at a more divergent point in its phylogenetic tree than earlier in its development.

### MAPK pathway genes demonstrate accumulation of non-synonymous variants

**Fig. 5:**
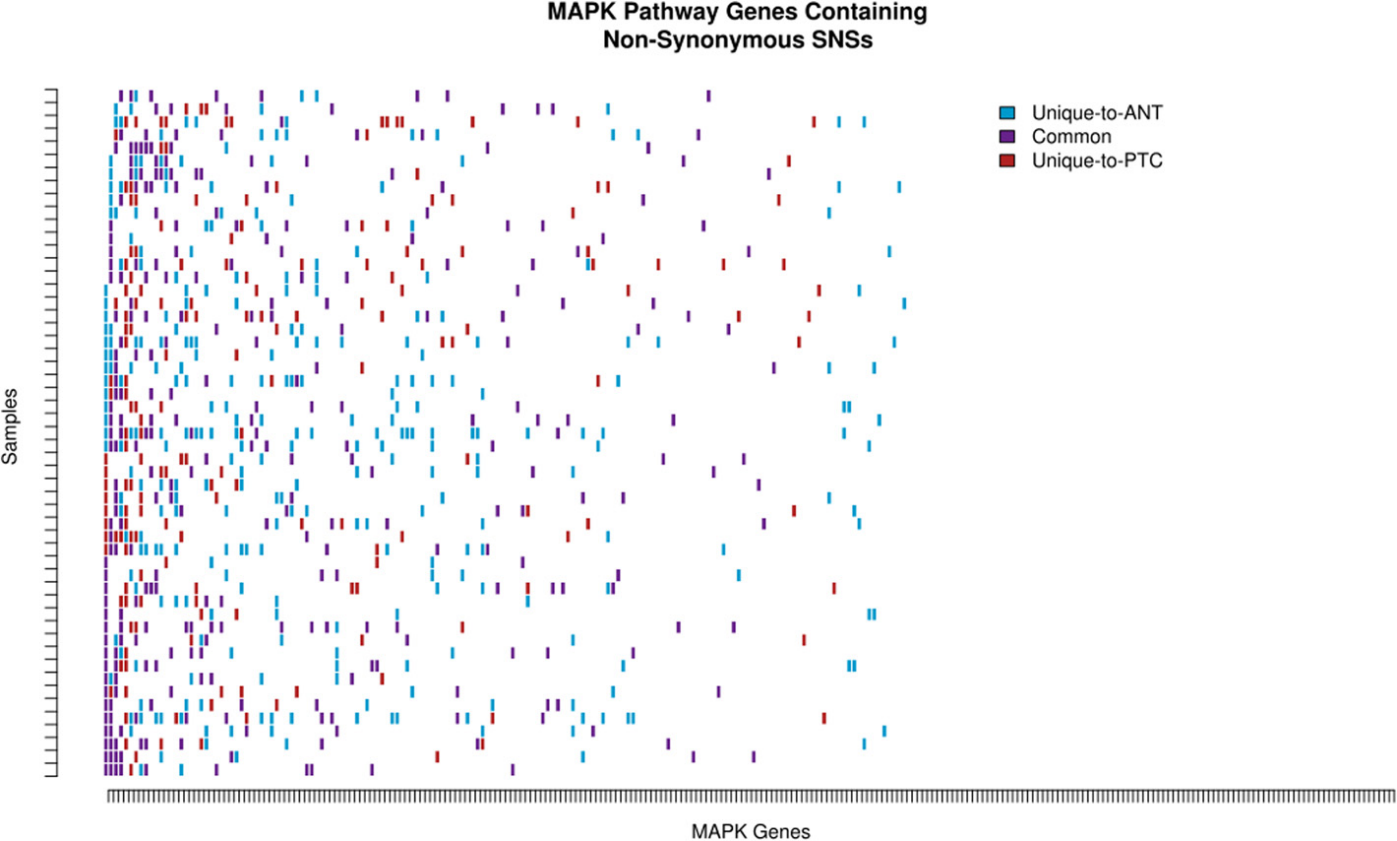
Non-synonymous SNSs in the MAPK Pathway. A number of recurrently altered genes are identified throughout the cohort, particularly MAP3K4, CACNA1B, and PAK2. Each of the BRAF-mutated samples contains at least one non-synonymous SNS in an additional MAPK pathway gene in the Common Subset (gene and sample IDs provided in Additional file 7: Table S2)

To identify potential functional impacts of the observed shift in mutational spectra, pathway-based enrichment analysis was performed on the sets of genes containing non-synonymous SNSs in the Unique-to-ANT, Common, and Unique-to-PTC subsets. Although their FDR-corrected q-values did not reach statistical significance, four pathways with *p*-values < 0.01 were related to MAPK (Additional file 7: Table S2). Specific examination of MAPK pathway genes in the primary sequencing data revealed several recurrently altered genes in the cohort [25, 26]. Many of the MAPK SNSs appeared in the Common subset, signifying their presence prior to divergence of malignant from non-malignant clones (Fig. 5, Additional file 8: Table S3). Thirty-eight samples (70.4 %) contained a MAP3K4 variant, seventeen of which occurred in the Common subset. This is in contrast to the twenty-three BRAF variants, all of which were in the Unique-to-PTC subset. Interestingly, each BRAF-mutated sample harbored non-synonymous MAPK pathway SNSs in the Common subset, frequently in MAP3K4, CACNA1B, and PAK2. This observation raises the question as to whether BRAF mutation represents a damaging second hit to an already vulnerable pathway. On average, each sample carried 5.9, 5.7, and 3.4 non-synonymous SNSs among MAPK pathway genes in the Unique-to-ANT, Common, and Unique-to-PTC subsets, respectively. Taken together, these findings suggest that non-synonymous SNSs in the MAPK pathway are frequent events, and that a subset of genes within the pathway is preferentially altered in the thyroid of PTC patients.

## Conclusions

The genome is a dynamic entity, constantly accruing changes that are subject to selective forces from diverse micro-environments. The true germline should be thought of as that present in the zygote, with subsequent cell divisions introducing change via imperfect DNA replication. Cells following variable differentiation pathways will therefore share a set of variants that derives from their most recent common ancestor cell. The accumulated evidence for this evolutionary framework is substantial and application of its principles will be critical in advancing understanding of the genome in health and disease. The current study, comparing thyroid tumor tissue, non-tumor thyroid tissue, and blood from single individuals demonstrates a great deal of heterogeneity within the genomic variants characterizing any given patient’s tissue types and provides further support for the somatic evolution model. These findings have potentially far-reaching implications regarding the common practice of using blood as the non-tumor control in attempting to identify tumor-specific mutations. The degree of diversity introduced through somatic evolution at every branch-point in differentiation results in vast tissue-specific variability, helping to explain a high incidence of false positives among proposed tumor driver candidates.

Previous analysis of mutational spectra in multiple tumor types demonstrated a predominance of C-to-T transitions in thyroid cancer [27]. Taken in their entirety, the current PTC cohort confirms this mutational pattern but demonstrates an evolutionary time-line wherein later changes are dominated by an alternative, potentially damaging, signature. This variation in SNS type at different points in thyroid development suggests that although individual SNSs may occur stochastically, there exist unifying forces likely informed by the micro-environment of differentiated thyroid as a whole or in subsections of the gland. For instance, binding of triiodothyronine to thyroid hormone receptor B in cell culture and in mice has been shown to increase levels of reactive oxygen species (ROS). ROS generated via normal aerobic metabolism, inflammation, and exposure to ionizing radiation are also known to cause DNA damage, preferentially at guanine residues resulting in 8-oxoguanine (8-oxoG) and thymine glycol [28]. Indeed, thyrocytes are uniquely sensitive to ionizing radiation, an established risk factor for PTC [1]. Chromosomal regions with high 8-oxoG density co-localize with high rates of single nucleotide polymorphism [29–31]. Furthermore, it has long been known that 8-oxoG pairs with adenine, resulting in C-to-A transversions [32]. Accumulation of 8-oxoG occurs in Mth1/Ogg1/Muthy triple knockout mice which develop various types of tumors that are heavily enriched for C-to-A transversions [33]. This series of observations provides a plausible mechanism through which the micro-environment of the thyroid could cause the observed shift in mutational spectra. Interestingly, multifocality is present in 15-25 % of patients, with tumor foci that seem to occur independently [34]. This clinical observation is consistent with the evolutionary model of PTC, in which independent multifocal tumors may arise in the background of genetic predisposition acquired during thyroidogenesis.

The current study represents a first step toward elucidating broader organizing principles that may underlie the individual genomic events previously associated with subsets of PTCs. The observed global shift in genomic variants causes increased likelihood of deleterious change to the encoded proteins in both malignant and non-malignant portions of the thyroid, leading to the postulation that stochastic factors (exact SNS location, order in which variants occur, micro-environment at the time of inception) may influence the potential for any given change to represent a true tumor driver. Further interrogation of the PTC genome should focus on identifying variants that cause evolutionary branch-points, as well as on elucidating the order of variant accrual. Such could be achieved through multiple sampling of individual PTCs, repeat sampling at various time-points, and via computational modeling, all integrated with longitudinal clinical data. Experience in multiple cancer types has shown that therapies targeting single tumor drivers will almost inevitably result in the selection and propagation of clones driven by alternative pathways. By coupling an expanded knowledge of the global trends underlying genomic evolution with a comprehensive catalog of driver variants placed in their evolutionary context, one could instead envision a therapeutic strategy aimed at modulating a dynamic system in order to minimize progression and focus on chronic management.

## Additional files

Additional file 1: Table S1. Shows Patient Demographic and Clinical Information. (XLSX 12 kb) Additional file 2: Figure S1. Shows the Transition-to-Transversion ratios. The Common Subset demonstrates a higher Tr:Tv than both the Unique-to-ANT and Unique-to-PTC subsets. (TIFF 1993 kb) Additional file 3: Figure S2. Shows the mutational spectra using alternate SNS filtering methods demonstrate that the shift in predominant SNS from C-to-T to C-to-A persists regardless of the level of SNS filtering: a) unfiltered; b) filtered to remove variants contained in dbSNP, build 135; and c) filtered to remove both dbSNP build 135 and 1000 Genomes variants. (TIF 115 kb) Additional file 4: Figure S3. Describes mutations at the level of the Genetic Code. Plotting the functional consequence of each base change upon translation of the sixty-four triplet codons in the genetic code shows that 34.4 % of potential C-to-T transitions result in synonymous change compared to only 19.8 % of C-to-A transversions. (TIFF 1550 kb) Additional file 5: Figure S4. Demonstrates the functional consequence of each SNS. Relative to the Common subset and the 1000 Genomes cohort, the increased frequency of C-to-A transversions in the Unique-to-PTC and Unique-to-ANT subsets show an accompanying increase in the frequency of non-synonymous change (*p* < 0.0001). (TIFF 2025 kb) Additional file 6: Figure S5. Demonstrates predicted impact of SNS on protein function. Based upon computational prediction with MutationTaster, RadialSVM, SIFT, LR. The observed shift toward increased numbers of C-to-A transversions is accompanied by an increased likelihood of accruing damaging variants in both the Unique-to-PTC and Unique-to-ANT Subsets across multiple algorithms (all *p*-values < 0.0001). (TIFF 3828 kb) Additional file 7: Table S2. Shows ConsensusPathDB Pathway analysis. (XLSX 23 kb) Additional file 8: Table S3. Shows Single Nucleotide Substitutions in the MAPK Pathway. (XLSX 43 kb)

## Abbreviations

ANT: Adjacent Non-tumor Thyroid
PTC: Papillary Thyroid Cancer
QS: Quality Score
ROS: Reactive Oxygen Species
SNS: Single Nucleotide Substitution
Tr: Transition
Tv: Transversion
