# Supplementary figures and images for: Shifting patterns of genomic variation in the somatic evolution of papillary thyroid carcinoma
Source: BMC Cancer. 2016 Aug 18;16:646. doi: 10.1186/s12885-016-2665-7 (PMC4989347; doi:10.1186/s12885-016-2665-7)

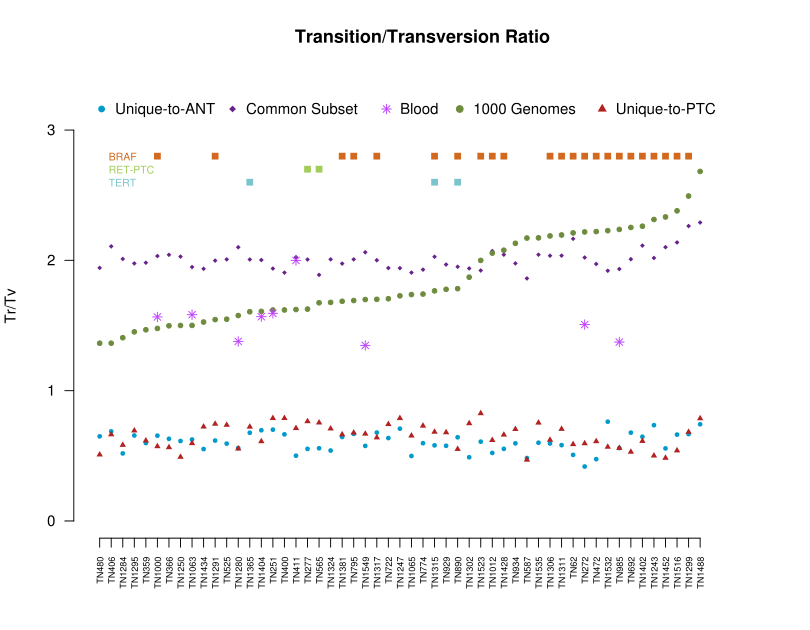

Supplement: Additional file 2: Figure S1. — Shows the Transition-to-Transversion ratios. The Common Subset demonstrates a higher Tr:Tv than both the Unique-to-ANT and Unique-to-PTC subsets. (TIFF 1993 kb) [file 12885_2016_2665_MOESM2_ESM.tiff]

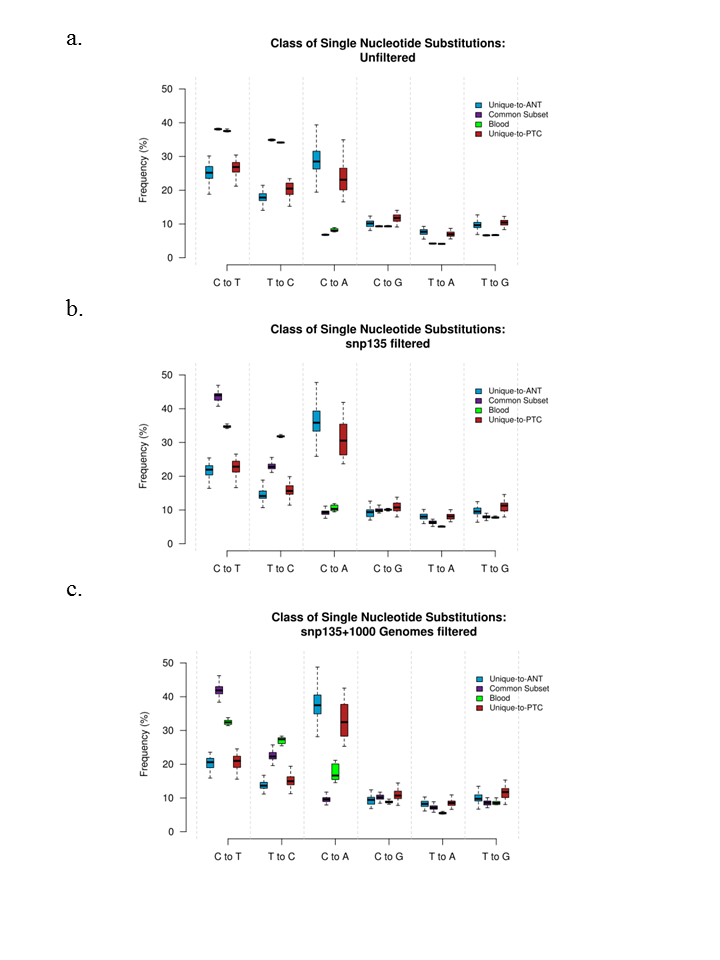

Supplement: Additional file 3: Figure S2. — Shows the mutational spectra using alternate SNS filtering methods demonstrate that the shift in predominant SNS from C-to-T to C-to-A persists regardless of the level of SNS filtering: a) unfiltered; b) filtered to remove variants contained in dbSNP, build 135; and c) filtered to remove both dbSNP build 135 and 1000 Genomes variants. (TIF 115 kb) [file 12885_2016_2665_MOESM3_ESM.tif]

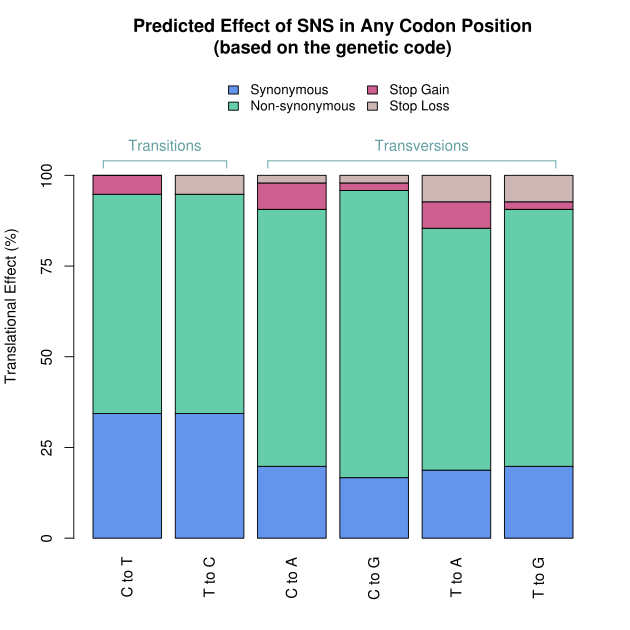

Supplement: Additional file 4: Figure S3. — Describes mutations at the level of the Genetic Code. Plotting the functional consequence of each base change upon translation of the sixty-four triplet codons in the genetic code shows that 34.4 % of potential C-to-T transitions result in synonymous change compared to only 19.8 % of C-to-A transversions. (TIFF 1550 kb) [file 12885_2016_2665_MOESM4_ESM.tiff]

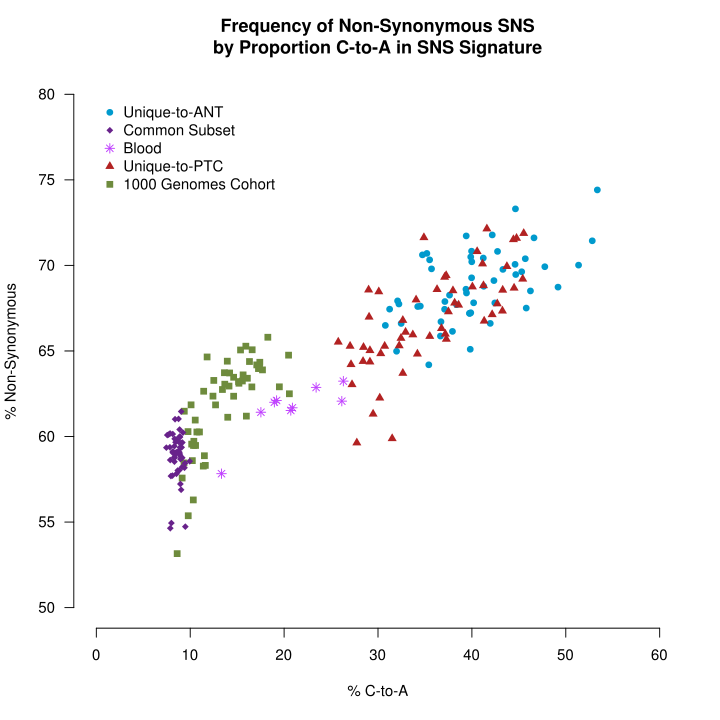

Supplement: Additional file 5: Figure S4. — Demonstrates the functional consequence of each SNS. Relative to the Common subset and the 1000 Genomes cohort, the increased frequency of C-to-A transversions in the Unique-to-PTC and Unique-to-ANT subsets show an accompanying increase in the frequency of non-synonymous change (p < 0.0001). (TIFF 2025 kb) [file 12885_2016_2665_MOESM5_ESM.tiff]

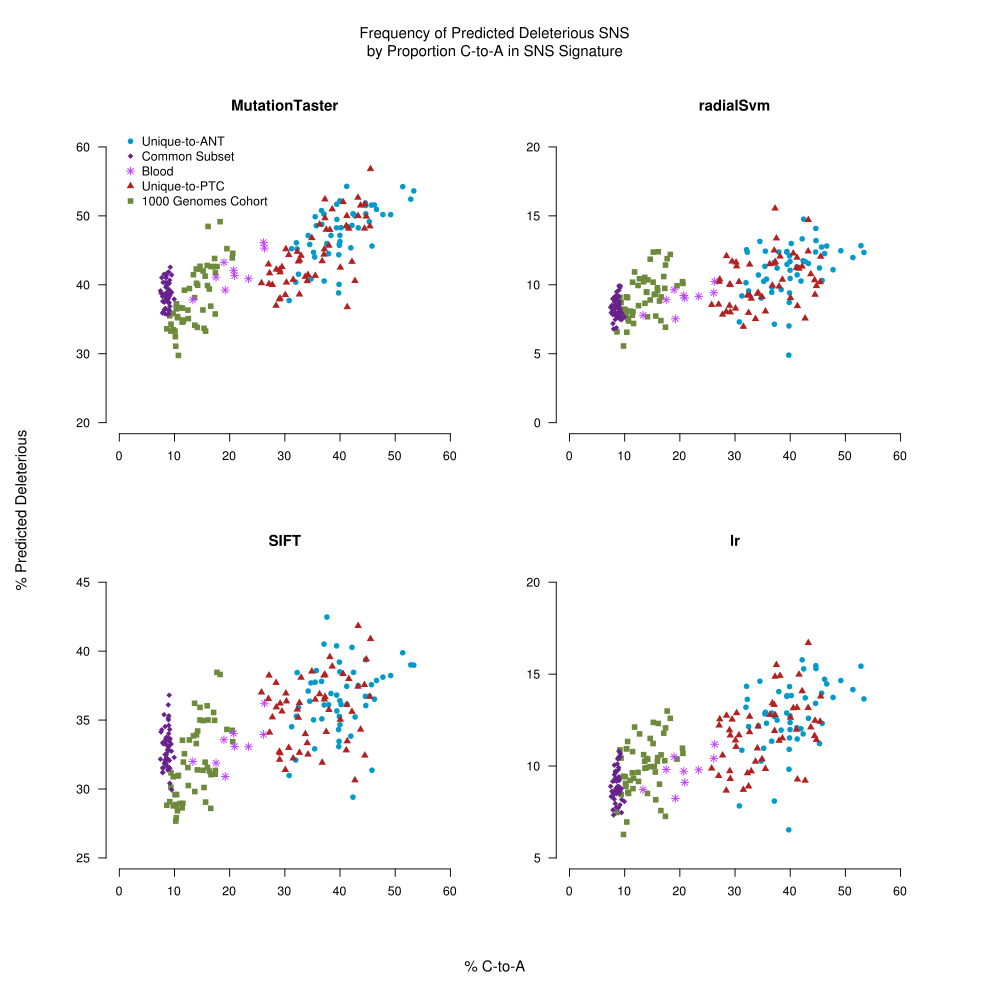

Supplement: Additional file 6: Figure S5. — Demonstrates predicted impact of SNS on protein function. Based upon computational prediction with MutationTaster, RadialSVM, SIFT, LR. The observed shift toward increased numbers of C-to-A transversions is accompanied by an increased likelihood of accruing damaging variants in both the Unique-to-PTC and Unique-to-ANT Subsets across multiple algorithms (all p-values < 0.0001). (TIFF 3828 kb) [file 12885_2016_2665_MOESM6_ESM.tiff]
